# Ultrasensitive and Tunable Achiral Metamaterial Substrates as Nanobiosensors for Enantiomer Detection

**DOI:** 10.1021/acsami.5c14316

**Published:** 2025-11-13

**Authors:** Maryam Mirahmadi, Ali Douaki, Vincenzo Caligiuri, Denis Garoli, Roman Krahne

**Affiliations:** † Optoelectronics, 121451 Istituto Italiano di Tecnologia (IIT), via Morego 30, 16163 Genova, Italy; ‡ Department of Chemistry and Industrial Chemistry, University of Genoa, via Dodecaneso 31, 16146 Genova, Italy; § Dipartimento di Science e Metodi per l’Ingegneria, Università degli Studi di Modena e Reggio Emilia, Viale Amendola 2, 14122 Reggio Emilia, Italy; ∥ Dipartimento di Fisica, 18950 Universitá della Calabria, via P. Bucci 33b, 87036 Rende, CS, Italy; ⊥ Consiglio Nazionale delle Ricerche − Istituto di Nanotecnologia (CNR-Nanotec), via P. Bucci 33c, 87036 Rende, Italy

**Keywords:** circular dichroism, chirality enhancement, enantiomer detection, plasmonic nanohole array, photonic cavity, biosensing

## Abstract

Distinguishing the chirality of biomolecules is of fundamental importance in biophysics and pharmaceutics. Circular dichroism (CD) spectroscopy provides a noninvasive approach to distinguish right- and left-handed enantiomers and can offer valuable insight into the structure of the investigated molecules. However, the intrinsic CD signal of biomolecules is often weak and typically resides in the ultraviolet spectral range, for which optical components are costly. Therefore, a tunable platform that boosts the CD signal of analytes in the optimal spectral range for the desired application would be an ideal solution. We combine tilted plasmonic nanohole arrays with ultrathin photonic cavities in our metamaterial substrates to achieve very high CD signal enhancement. In this approach, the spectral region with a high CD signal can be tailored by the geometric parameters of the array and cavity. In a tilted geometry, achiral structures mimic chiral behavior, offering an interesting alternative to inherently chiral structures. Our work highlights the role of near-field optical chirality and of the chirality enhancement factor, χ, in boosting the CD signal. To combine plasmonic and photonic effects for CD enhancement, we integrate the nanohole array as a top layer in a metal–dielectric–metal cavity structure. This metamaterial design strongly amplifies the electromagnetic near field and, in particular, its asymmetry. From both experimental and numerical results, we obtain another 10-fold increase in the χ factor, leading to a 50-fold enhancement compared to the bare biolayer on glass. With our robust and intrinsically achiral plasmonic and photonic metamaterial structures, we introduce a versatile platform for enantiomer discrimination and nanobiosensing applications that allows for spectral tuning of the operational resonance band via the geometry of the lattice and cavity.

## Graphical abstract

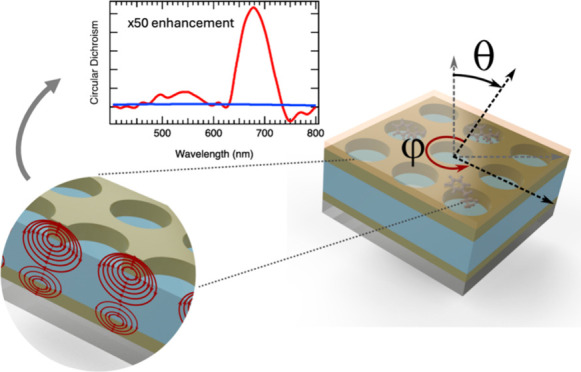

## Introduction

Chirality is a widespread phenomenon in nature, playing a fundamental role in living organisms. Today, we know the impact of chiral biomolecules such as proteins, amino acids, and carbohydrates on the functionality of living organisms. This understanding highlights the importance of distinguishing between enantiomers, particularly in biology and pharmaceutics, where safety and cost efficiency are paramount. Enantiomers are nonsuperimposable mirror-image molecules with distinct biological or chemical properties,^1^ and opposite enantiomers can be distinguished by circular dichroism (CD) measurements because they exhibit preferential absorption of left/right circularly polarized light (L/R-CPL).

However, CD spectroscopy faces a key challenge: the detection of weak chiroptical signals due to limited chirality-induced interactions with plane wave light fields.^2^ With the advent of plasmonics and metamaterials, exploring new solutions to enhance these weak chirality effects has been an area of extensive research. Nanostructures and metamaterials have been designed to replicate chiral molecule properties, enabling light polarization control and CD enhancement across specific wavelengths. For instance, giant circular dichroism signals have been reported in both the visible and near-infrared (NIR) spectral ranges.^3,4^

Researchers have explored a range of periodic structures as substrates, including 3D nanohelix arrays,^5^ twisted cross rods,^6^ racemic nanoplasmonic arrays,^7^ chiral assemblies of nanoparticles,^8^ and achiral plasmonic nanostructures.^9^ Other approaches include prism-coupled surface plasmon systems at planar silver–solution interfaces for CD enhancement,^10^ theoretical modeling of plasmonic CD via Coulombic and electromagnetic interactions in chiral nanoparticle assemblies,^11^ and experimental and numerical demonstrations of tunable CD using vertically aligned gold nanorod arrays embedded with chiral mercury sulfide nanocrystals in a polymer matrix, enabling strong CD enhancement in the visible range.^12^ Meniscus-guided self-assembly of achiral CdS, CdSe, and CdTe nanoclusters into helical domains results in giant exciton-coupled CD,^13^ and strategies leveraging biomolecular functionalization of metal nanoparticles facilitate chirality transfer,^14^ for example CD of chiral Au nanorod seeds.^15^ Numerical calculations show that a gain medium surrounding twisted gold nanorod dimers enables tunable, orders-of-magnitude enhancement of circular dichroism.^16^ Additionally, attachment of chiral mercury sulfide nanocrystals to amorphous selenium nanospheres yields a 5-fold enhancement of visible circular dichroism via Mie resonances.^17^ Numerical studies reveal that chiral medium patches placed in plasmonic gap antenna hot spots can achieve up to 750-fold circular dichroism enhancement, surpassing chiral dimer designs.^18^

While most research emphasizes the intrinsic chiroptical response of substrates, the relationship between these responses and CD signals in the presence of biolayers remains insufficiently explored. In biosensing applications, the emphasis should be on the total CD response of substrate-supported biolayers rather than isolating the substrate’s contribution alone. Several studies have investigated CD enhancement in the presence of biolayers through comparative analysis. Leite et al. reported CD enhancement in the far-UV regime using Al gammadion arrays, however, this was only sufficient to lift signals just above the noise level for <10 nm tyrosine films, implying an enhancement of less than 5-fold.^19^ In plasmonic nanorod metamaterials, a 2-fold enhancement of circular dichroism was observed for embedded chiral mercury sulfide nanocrystals.^12^ Mohammadi et al. analytically modeled and experimentally demonstrated CD enhancement for thin chiral layers using dielectric metasurfaces, reporting enhancement values up to ∼15, utilizing accessible superchiral near-fields driven by tailored electric and magnetic resonances.^20^ Venturi et al. predicted a CD enhancement factor of 20 using surface plasmon polaritons in Kretschmann and Otto configurations for dilute chiral drug solutions,^10^ while García-Guirado et al. demonstrated up to 60-fold enhancement in experiments and 170 in simulations using racemic gammadion arrays for enantiomer-selective sensing in the visible range.^21^ Vázquez-Guardado and Chanda reported an achiral plasmonic system that generates pure superchiral near fields with zero far-field circular dichroism, enabling background-free molecular chirality detection. They achieved approximately 4 orders of magnitude enhancement in the asymmetry factor g for vibrational circular dichroism (VCD) sensitivity in low-volume chiral analytes, highlighting the system’s potential for ultrasensitive biosensing.^22^ Wang et al. demonstrate an extrinsic chiral plasmonic sensor based on achiral gold nanohole arrays at oblique incidence, where adding l- or d-phenylalanine yields opposite g-factor shifts and a g-factor change of 0.027 near 750 nm compared to 0.001 for the pure chiral medium.^23^

In this work, we use achiral gold nanohole arrays whose resonance band could be spectrally tuned by tailoring their geometric parameters, providing a flexible platform for enhancing circular dichroism signals through extrinsic chirality. Our analysis shows that the observed CD enhancement arises from chiral near fields that can be characterized by the chirality enhancement factor (χ), which encompasses both biolayer and substrate, and integrates electric and magnetic dipolar resonances. In our approach, we generate extrinsic chirality by tilting an achiral nanostructured substrate with respect to the incident light, which circumvents the need for inherently chiral geometries and offers tunable control over the chiroptical response.

The angular tilting of the incident wave introduces in-plane components to the wavevector, defined as *k*_*x*_ = *k*_0_sin­(θ)­cos­(φ), *k*_*y*_ = *k*_0_sin­(θ)­sin­(φ), where *k*_0_ = 2π/λ is the magnitude of the incident wavevector. These components play a critical role in satisfying the momentum-matching condition required for the excitation of surface plasmon polaritons (SPPs) on periodic nanostructures, introducing in-plane momentum via oblique incidence that enables efficient plasmonic coupling and breaks the symmetry of the excitation field relative to the nanostructure.^24^ This symmetry breaking results in asymmetric coupling strengths for RCP and LCP light, leading to differential field distributions and consequently distinct transmission or absorption pathways. The resulting difference in transmittance (Δ*T*) gives rise to a measurable CD signal, despite the intrinsically achiral underlying structure.^25,26^

Our study is structured as follows: (i) we fabricate and investigate the CD response of a tilted single gold nanohole array as a substrate, (ii) we study the CD response of l-phenylalanine as a chiral biolayer in two conditions—isolated and placed on the single gold nanohole array, and (iii) we then combine plasmonic and photonic resonator effects by fabricating a metal–dielectric–metal (MDM) metamaterial structure composed of Au/Al_2_O_3_/Au with nanoholes in the top gold layer, and subsequently deposit an l-phenylalanine biolayer on top. This proves to be a highly promising platform for achieving strong CD signal, even compared to that obtained using a single gold nanohole layer as substrate.

## Results and Discussion

In this study, we investigated the CD response of two types of fabricated samples. The first was a single-layer gold nanohole array, presented in the scanning electron microscopy (SEM) image in Figure 1a and sketched in Figure 1b. The second was a multilayer Au/Al_2_O_3_/Au nanohole structure that will be discussed later and which is depicted in Figure 5a. Both substrates were coated with a thin layer of the chiral molecule l-phenylalanine.

**Figure 1:**
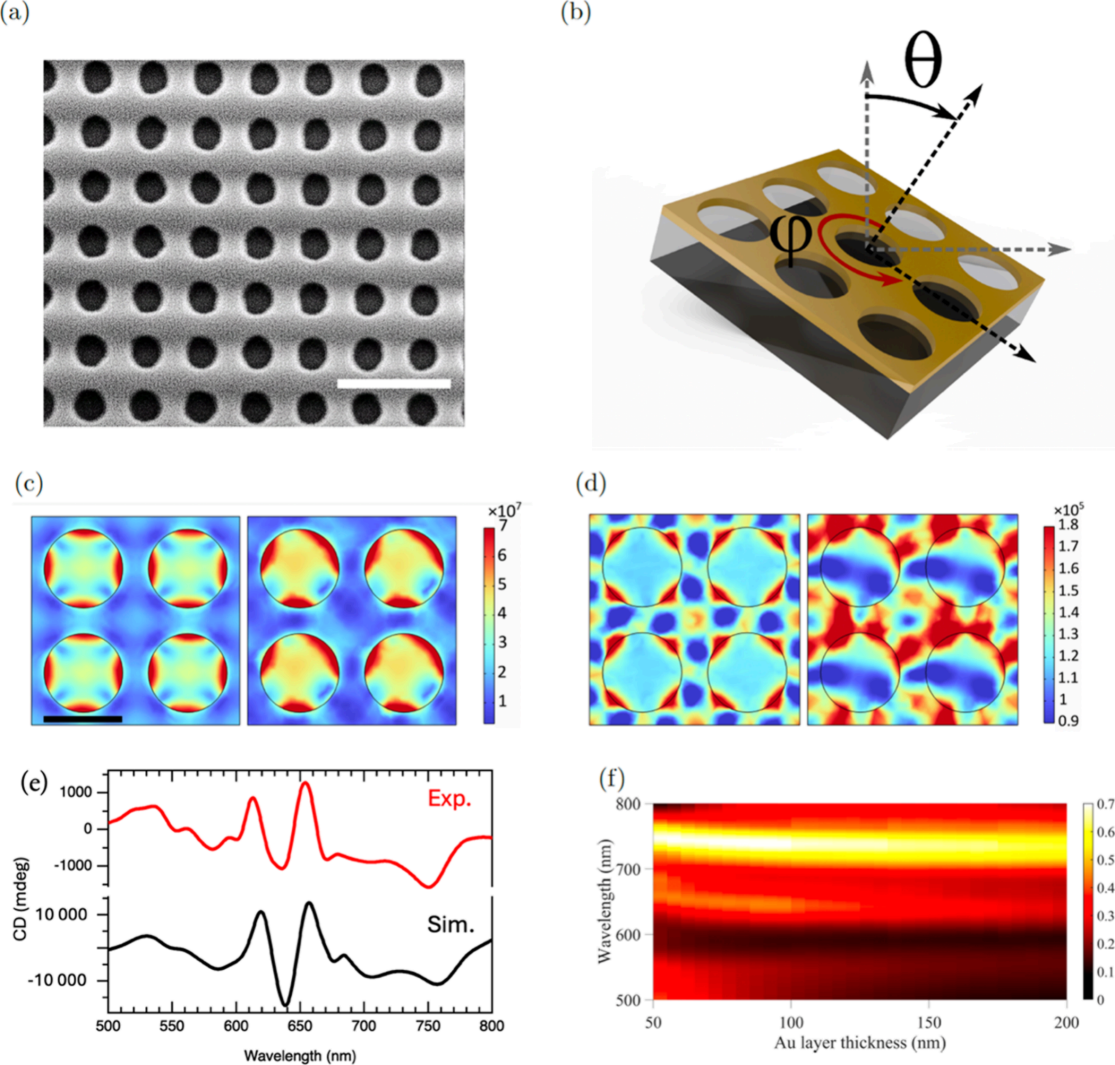
(a) Scanning electron microscope (SEM) image displaying the nanohole gold layer with a thickness of 80 nm on a glass substrate with a size of 12 × 12 mm^2^. The radius of the nanoholes is 200 nm and lattice constant is 530 nm. Scale bar is 1 μm. (b) Schematic illustration of a nanohole gold layer sample under oblique incidence light (θ and φ, where θ denoted the tilt angle between the incident wave vector k and the normal vector of the surface, and φ represents the azimuthal rotation angle of the plane of incidence). (c) Calculated electric and (d) magnetic field distributions. The left panel corresponds to perpendicular incidence (θ = 0° and φ = 0°), displaying mirror-symmetric patterns at the transmission resonance wavelength of 725 nm. The right panel represents the enhanced asymmetric electric field distribution under oblique incidence (θ = 10° and φ = 30°). Scale bar is 500 nm for all panels. (e) CD response of the gold nanohole array: experimental results (red curve) compared with simulation data (black curve). (f) Color map of transmission as a function of gold thickness and wavelength. The plot highlights regions of significant transmission, with a pronounced bright band observed around 80 nm of gold thickness in the 700–750 nm wavelength range.

### Circular Dichroism Measurements

The CD response of the samples was measured in transmission using a custom-designed sample holder integrated into the optical transmission setup (see Supporting Information for details, Figure S1). The holder was designed and manufactured to enable two-axis sample orientation for the measurements by equipping it with dual rotation axes, as illustrated in Figure 1b. The CD response, measured as the transmission difference between right- and left-handed circularly polarized light, was determined using eq 1, where *I*_R_ and *I*_L_ represent the transmitted intensities for right and left circularly polarized light, respectively.^9^

$$\mathrm{CD}=\tan^{-1}\left(\frac{I_{R}-I_{L}}{I_{R}+I_{L}}\right)$$ 1

Figure 1c,d show the electric and magnetic field distributions at resonance wavelength of 725 nm for the perpendicular and tilted configurations with θ = 10° (out of plane) and φ = 30° (in plane). This analysis demonstrates how tilting the sample, as depicted in Figure 1b, disrupts its structural symmetry and induces chiroptical activity. The CD spectra of the fabricated gold nanohole structure at these angles exhibit peaks at 619 and 656 nm, as shown by the red curve in Figure 1e. The simulated CD responses at the same angular orientations, represented by the black curve in Figure 1e, align well with the experimental results, with corresponding valleys and peaks in both signals. The optimum thickness of the gold layer was determined numerically. The transmission colormap in Figure 1f reveals that a structure with an 80 nm thick gold layer exhibits a pronounced transmission peak in the 700–750 nm wavelength range, which coincides with the range of interest for CD enhancement. A second weaker transmission band can be observed around 640 nm for layer thicknesses up to 100 nm.

### Finite Elements Simulations

The simulations were performed using COMSOL Multiphysics software with the Electromagnetic Waves, Frequency Domain physics interface in a 3D configuration. Figure 2a–f presents a comparison of CD measurements and simulations for varying φ and constant θ, demonstrating very good agreement. For both simulation and experimental data, increasing the φ angle causes a shift in the CD response, and the magnitude starts to increase. The dashed curves represent negative φ angles, which exhibit similar behavior.

**Figure 2:**
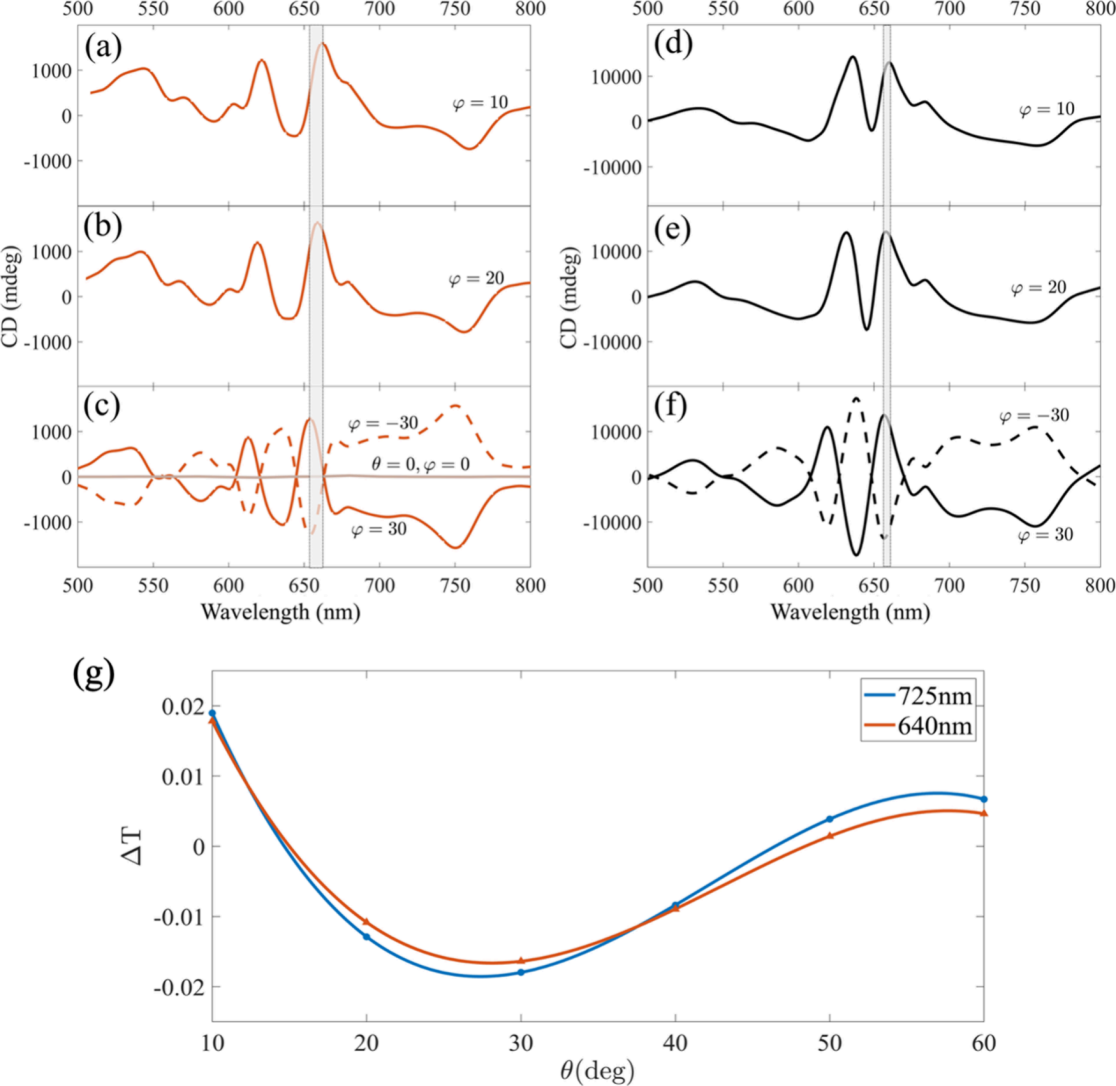
(a–g) Angular dependence of CD in gold nanohole arrays. (a–c) Experimental and (d–f) calculated angle-dependent CD signals of a gold nanohole array as a function of azimuthal angle φ and constant θ = 10°. Dashed curves in (c) and (f) represent negative φ angles, and the range around 650 nm is marked to emphasize the small shift of the maximum with increasing φ. (g) Parametric plot showing the simulated transmission difference (Δ*T*) between right- and left-circularly polarized light versus incident angle of θ at resonant wavelengths of 725 and 640 nm in the transmission spectra. The parabolic trend observed from 10° to 60° highlights the angular dependence of chiroptical response in the tilted nanostructure. The maximum Δ*T* occurs at θ ≈ 30°, indicating an optimal tilt angle for enhancing CD signal.

To investigate how the incident angle θ influences the chiroptical response, we start from the general plane wave equations, expressed as $\vec{E}={\vec{E}}_{0}e^{i(\vec{k}\times \vec{r})}$ . Upon tilting the sample, the wavevector $\vec{k}$ is no longer purely in the *z*-direction but gains in-plane components *k*_*x*_ and *k*_*y*_, which are functions of the polar angle θ and azimuthal angle φ.^21,22^ Specifically, $\vec{k}$ = *k*_0_(sinθcosφ,sinθsinφ,cosθ), which renders the phase term $\vec{k}\times \vec{r}$ explicitly dependent on the tilt angles. This angular dependence alters the interaction between light and the nanostructure, modifying the phase and amplitude of the transmitted field differently for right- and left-circularly polarized light.

By calculating the square of the electric field magnitude, we can determine the transmittance as $T\propto |\vec{E}{|}^{2}$ . Substituting this into the definition of Δ*T*, the difference in transmission between right- and left circularly polarized light-leads to an expression where Δ*T* depends on the incidence angle θ. This theoretical approach predicts a parabolic trend in Δ*T* as a function of the incidence angle θ, consistent with the results obtained from our simulations in the range of 10°–60°, as shown in Figure 2g.

The differential transmission Δ*T* between right- and left-circularly polarized light was evaluated at wavelengths of 725 and 640 nm, which correspond to prominent resonance features in the transmission colormap (see Figure 1f). This result confirms that changing θ enhances Δ*T* and leads to measurable CD signals, indicating the emergence of extrinsic chirality. Notably, the maximum Δ*T* was observed at θ = 30°; however, due to the limited numerical aperture (NA) of the long working distance microscope objectives used in our setup (NA = 0.4), the experimental measurement of the CD response was constrained to tilt angles up to 10°. At larger tilt angles, the incident and transmitted beams deviate beyond the acceptance cone defined by the NA, resulting in partial light collection and reduced measurement accuracy.

### Simulation of an Isolated Biolayer

To gain an understanding of the combined system consisting of substrate and biolayer, we first discuss the properties of an isolated chiral biolayer. The chiral biolayer is considered as a slab with a thickness of *w*_b_, positioned in free space and characterized by the refractive index *n*_b_ and the Pasteur parameter κ_b_, which quantifies the material’s chirality.^27^ The wavevectors for the right circular polarized (RCP) and left circular polarized (LCP) waves are denoted as ±κ. To analytically compute the CD signal, the transmission amplitudes for the RCP and LCP excitations were determined separately at oblique incident light conditions using chiral media in COMSOL software. The equations of the software were modified as eqs 2 and 3 to effectively model the chirality. In the equations, $\overset{↼}{H}$ represents the magnetic field, and $\overset{↼}{B}$ denotes the magnetic flux density. Parameters ε_0_ and ε_r_ are the vacuum and relative permittivity, and μ is the permeability.^21^

$$\overset{↼}{D}=\varepsilon_{0}\varepsilon_{r}\overset{↼}{E}+i\kappa \overset{↼}{H}$$ 2

$$\overset{↼}{H}=\frac{\overset{↼}{B}}{\mu }+j\kappa E$$ 3

Figure 3a,b shows the CD of the simulated l-phenylalanine as a chiral biolayer with a thickness of 60 nm and optical properties of *n*_b_ = 1.6 and a Pasteur parameter of κ_b_ = (5 – 0.05*i*) × 10^–5^.^21^ We have investigated the UV-CD of the biolayer coatings on silica as well as the CD response of our plasmonic sensors. Figure 3a depicts the CD spectra of a biolayer in the UV/vis/NIR range, 200–800 nm for two separate coatings with l- and d-phenylalanine. CD spectra of both coatings of l- and d-phenylalanine reveal a main peak at 250 nm. Although the CD signal of the biolayer resides in the UV range, we design our sensor substrates for the Vis and NIR regions because sensing in the UV is more demanding concerning optical components. With our strategy of using substrates that enable tunable resonances in the Vis-NIR range, we develop a sensor that effectively captures the analyte’s response while ensuring straightforward compatibility with standard optical sensing. We show the full-wave simulations of both single l- and d-phenylalanine in Figure 3a. These calculated CD values in the visible range were then used to calculate and compare the magnitude of the enantiomeric-enhanced signals when the molecules were deposited on the sensors.

CDb=−tan−1[tanh(2k0μbIm{κb})] 4

We employed analytical calculations based on eq 4 to estimate the CD of the biolayer in the Vis-NIR spectral range that we target for sensing, and compared the results with simulations.^27^ Figure 3b shows good agreement between the two approaches.

**Figure 3:**
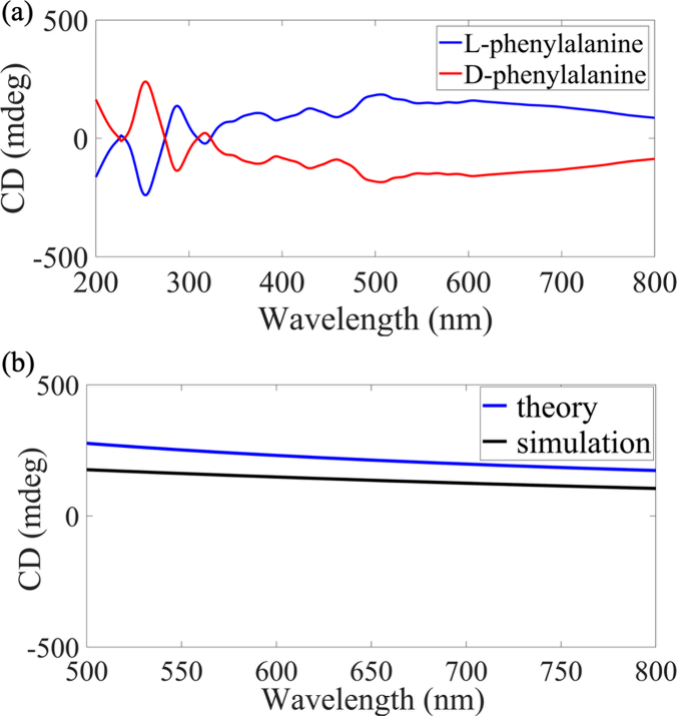
(a) Simulated CD spectra of the l-phenylalanine (blue curve) coatings and its enantiomer, d-phenylalanine (red curve), the 200–800 nm range, revealing a main peak at 250 nm. (b) Comparison of the CD response of an l-phenylalanine from simulated (black curve) and theoretical (blue curve) studies in visible range.

Figure 4a presents the gold nanohole array on a glass substrate, including the biolayer positioned on top, and Figure 4b displays the induced CD enhancement measured by tilting it within the optical setup. Thickness measurements by atomic force microscopy (AFM) and optical characterization of an l-phenylalanine film are reported in Figure S2. The red curve represents the CD signal of the nanohole array, while the blue curve corresponds to the CD response of the single biolayer of l-phenylalanine on silica glass. Remarkably, a 20-fold enhancement in CD was observed when the biolayer was on the tilted nanohole arrays. The overall CD response features a pronounced peak at 725 nm, accompanied by two valleys at approximately 715 and 735 nm. Moreover, COMSOL simulations for the nanohole array-biolayer configuration successfully reproduced these results, as shown by the black curve in Figure 4b. The small discrepancies in the CD signal between the experimental and simulation data result from imperfections intrinsic to experimental measurements, such as deviations in the precise geometry of the nanohole array, and collimation and angle of the optical light beam.

**Figure 4:**
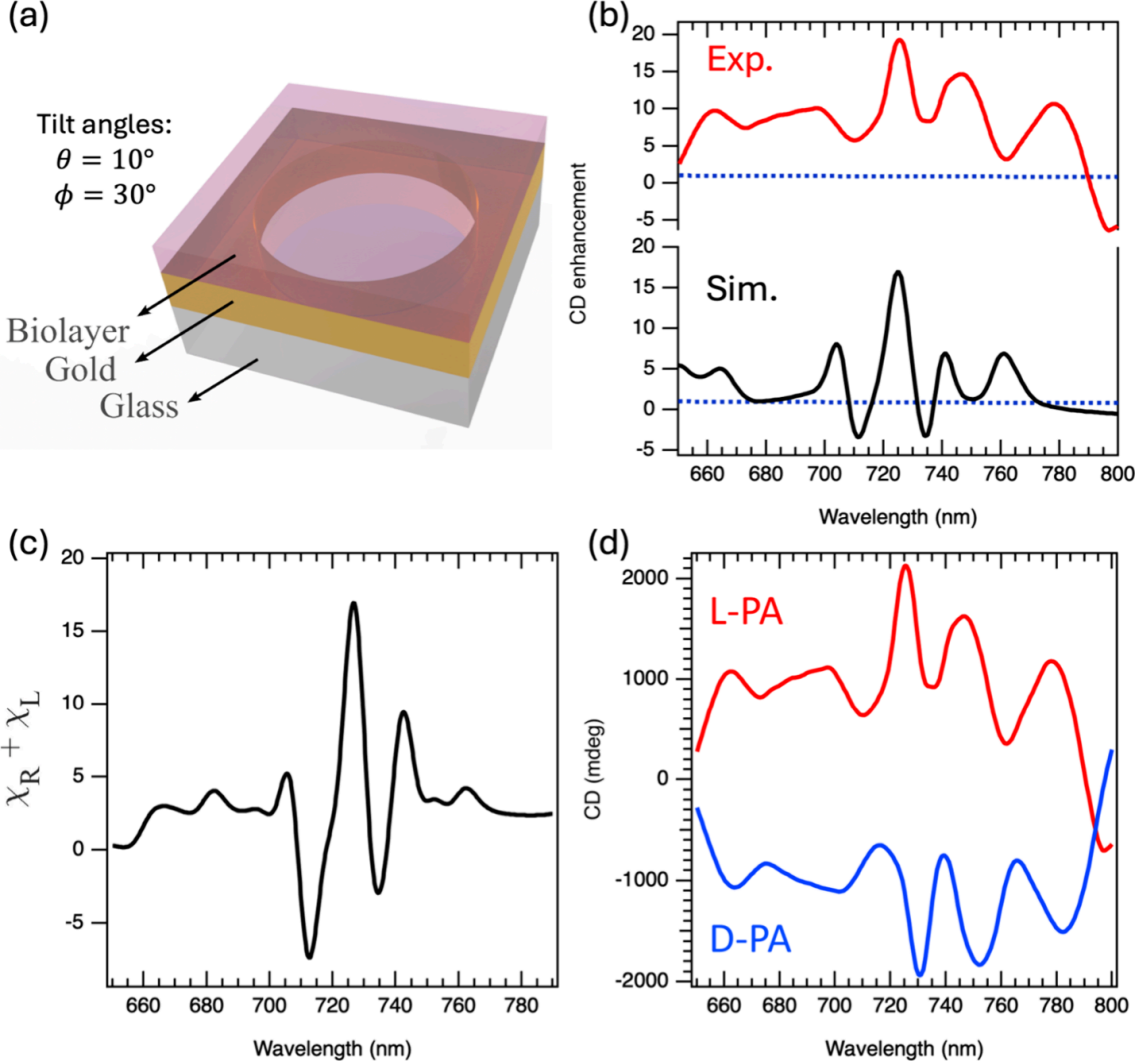
(a) Unit cell of the Au nanohole array on glass with biolayer. (b) Simulated (black curve) and experimental (red curve) CD spectra of the biolayer on gold nanohole array that exhibit valleys at the 715 and 735 nm, with a pronounced peak at 725 nm. The blue-dashed line in both charts illustrates the normalized CD response of a single biolayer of l-phenylalanine. (c) Analytically calculated χ, displaying valleys and peak at similar wavelengths as in the CD spectra in (b). (d) Experimental CD spectra of l-phenylalanine (L-PA-red) and d-phenylalanine (D-PA-blue).

In Figure 4b, the enantiomers on top of the nanohole array show strong CD signal in the range from 700 to 750 nm, distinct from the CD peak wavelength of the bare nanohole array (Figure 1e) but inside the transparency windows of nanohole substrate (Figure 1f). This happens due to the synergistic effect of the local optical chirality and the field enhancement of the nanohole array, which selectively amplify the residual CD of the enantiomers in the visible-NIR range. The signal enhancement stems from an imbalance in the near-field distribution of the gold nanohole array, where at certain frequencies the plasmonic modes interact more effectively with the enantiomers.

To identify the parameter that impacts the spectral maxima of the CD signal, we adopted an analytical approach, in which the CD was calculated following eq 5.^28^ The results were compared with those of finite element simulations, demonstrating good agreement, which validates our previous modeling approach.^29^

CDtotal=CDs+{k0wbIm{εb}2Re{εb}(FL−FR)+CDb2(χR+χL)}sech(4k0wsIm{κs}) 5

Equation 5 calculates the total CD of the structure, taking both the contribution of the nanohole array (CD_s_) and the biolayer (CD_b_) into account. κ_s_ and *w*_s_ represent the Pasteur parameter and thickness of the substrate, respectively. The parameter $F_{L(R)}$ represents the local intensity factors determined by ( $F_{p}^{\mathrm{loc}}=(|E{|}_{\mathrm{near}}^{2})/(|E{|}_{\mathrm{far}}^{2})$ ), and χ_L(R)_ denotes the chiral enhancement factors (χ_P_^loc^ = Im­{*E* × H*}_near_/Im­{*E* × H*}_far_).^25^ The χ factor is calculated by averaging χ across all points in the specified volume. Specifically, the near-field zone is defined as a volume of the same size as the chiral sample, immediately on top of the substrate, while the far field extends to the end of the physical simulation domain. We conducted full-wave range calculations of the CD signal for three configurations: a single nanohole array, a biolayer on glass, and a gold nanohole array-supported biolayer.

Equation 5 provides analytical insights into two influential factors: the sech coefficient and the χ factor. The former represents the chiral absorption of the substrate (*k*_0_ *w*_s_Im­{κ_s_}) in the CD spectroscopy and is weak, while χ plays a crucial role in enhancing CD of the biolayer as we demonstrate now. The spectra in Figure 4c show that the sum of the χ factors for RCP and LCP has two valleys at 715 and 735 nm and peak at 725 nm that coincide well with those of the CD enhancement in Figure 4b. Therefore, the χ factor is a key parameter for evaluating the potential of a substrate to function as a biosensor.

Figure 4b shows that the tilted nanohole array substrate provides very high CD enhancement of a factor of 20 at the resonance (725 nm) compared to a biolayer on bare glass (blue-dashed line). The CD spectra for both enantiomers, l- and d-phenylalanine, are shown in Figure 4d. Assuming that the two biolayers differ only in their CD signatures, which corresponds to opposite signs of the imaginary part of the Pasteur parameter, and reversing the handedness of the biolayer should result in a sign reversal of the CD signal, as clearly evident in the spectra.

### Chirality Enhancement via an Optimized Metal-Dielectric-Metal Structure

The substrate-mediated chiroptical enhancement can be further increased by combining the plasmonic nanohole array with an optical cavity. To this end, we designed and fabricated a metamaterial Metal–Dielectric–Metal (MDM) structure,^30,31^ in which the Au film with the nanohole array is the top metal layer, as illustrated in Figure 5a. This engineered nonchiral planar metasurface supports strong near-field electromagnetic effects, enabling significant amplification of optical chirality and CD.^32,33^ To find the optimal thickness of the dielectric layer, we simulated the transmission spectra of the MDM nanohole array structure over a range of Al_2_O_3_ thicknesses (Figure 5b). A thickness of 170 nm was selected, as it supports optical resonances in approximately the same spectral region as the single-layer gold nanohole substrate. To clarify the effect of the nanohole array within the MDM structure, we also compared these results with the transmission of a planar (nonpatterned) MDM thin film, as detailed in the Supporting Information (Figure S3). We expect synergies of the cavity and plasmonic enhancement effects, and this geometry allows for a straightforward comparison in enhancement efficiency with the nanohole array. Thus, the MDM structure consists of a 170 nm-thick Al_2_O_3_ layer sandwiched between a 30 nm bottom gold film and a 60 nm top gold film patterned with nanoholes. The transmission values presented in Figure 5b are normalized with respect to the transmitted power through the same boundary without any structure present, such that values greater than unity directly indicate constructive near-field enhancement and forward scattering owing to plasmonic Fabry–Pérot-like resonances and extraordinary optical transmission.^34,35^

**Figure 5:**
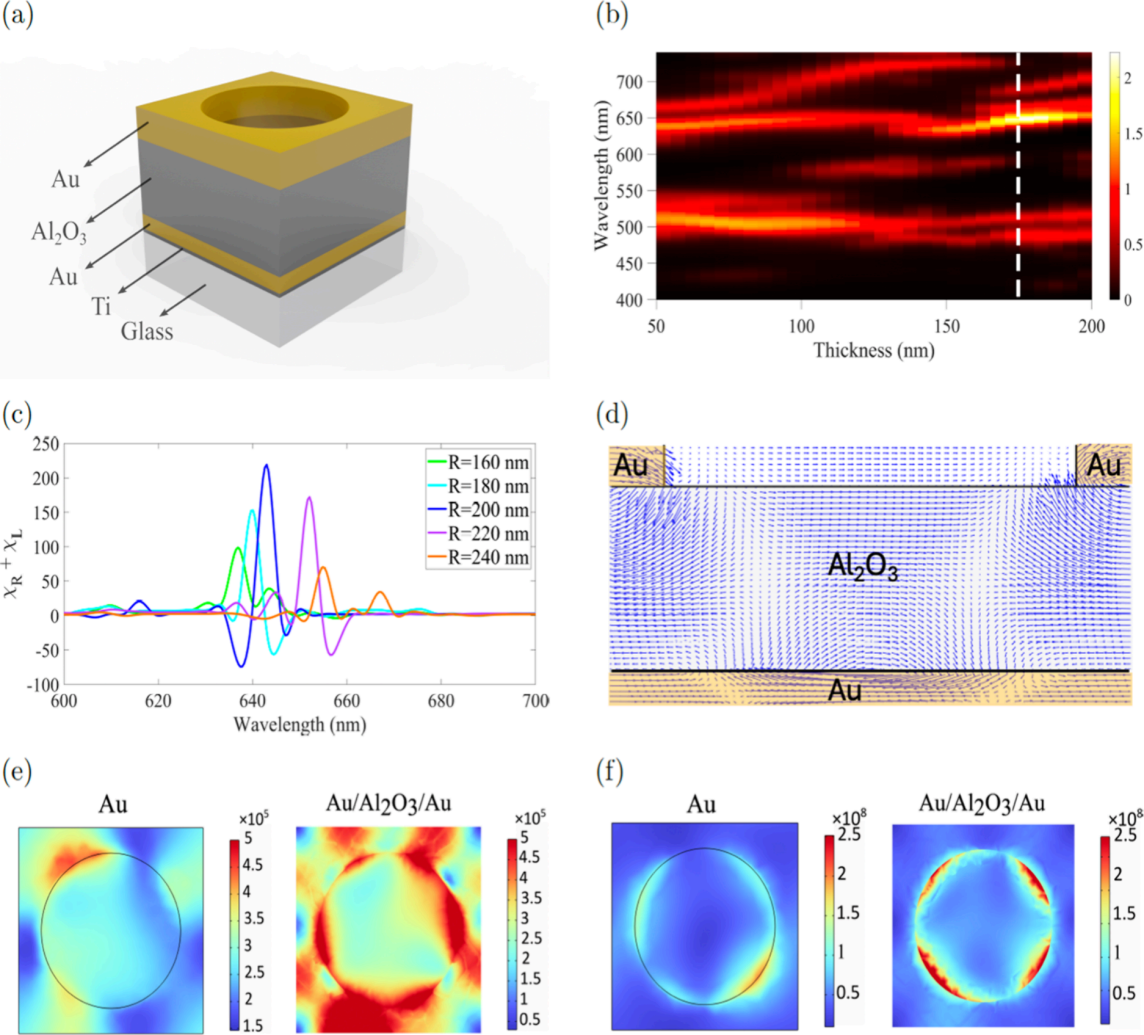
(a) Unit cell of the Au/Al_2_O_3_/Au nanohole array structure. (b) Colormap of simulated transmission as a function of Al_2_O_3_ thickness and wavelength. The color scale was adjusted to [0–2] to enhance the contrast of the narrow transmission peaks associated with the MDM cavity. (c) Calculated average optical chirality enhancement factor (χ) for varying nanohole radii. The maximum χ occurs at a radius of 200 nm. (d) Snapshot of the displacement current distribution in the *yz*-plane for the Au/Al_2_O_3_/Au nanohole array structure at λ = 643 nm. (e) Magnetic and (f) electric field distributions at transmission resonance λ = 725 nm for a single-layer gold nanohole array (left) and at transmission resonance λ = 643 nm for the Au/Al_2_O_3_/Au nanohole array structure (right), under oblique illumination (θ = 10°, φ = 30°). Magnetic and electric field are in A/m and V/m, respectively.

For further optimization, we evaluated the factor χ with respect to the nanohole radii ranging from 120 to 250 nm (Figure 5c). The results revealed a maximum value χ at a radius of 200 nm. The enhancement of the χ value is attributed to the near-field enhancement induced by the MDM-nanohole array cavity, in particular with respect to the magnetic field at resonance. Simulation of the current density distribution, shown in Figure 5d (*ZY* plane cross section of the nanohole array unit cell), demonstrates the formation of closed-loop displacement patterns within the MDM layers. These loops generate localized magnetic dipole resonances, consistent with the Biot–Savart law. In contrast, the single-layer gold nanohole structure does not support such circulating displacement currents, indicating that its chiroptical response arises primarily from comparatively weaker electric-dipole interactions rather than the magnetic-dipole modes supported in the MDM-nanohole array architecture. As shown in Figure 5e,f, the electric and magnetic field distributions in the MDM-nanohole array structure are significantly stronger than those observed in the single-layer gold nanohole array.^36^

To experimentally validate the sensing capability of the MDM-nanohole array substrate (Figure 6a), a 60 nm-thick l-phenylalanine biolayer was deposited on both glass and the MDM-nanohole array substrates. CD measurements in Figure 6b show a ∼50-fold enhancement in CD signal when the biolayer was placed on the MDM-nanohole array substrate, compared to glass. Under similar conditions, the single-layer gold nanohole substrate exhibited a 20-fold CD enhancement. Simulations further predicted up to 80-fold CD amplification for the MDM-nanohole array configuration as shown in Figure 6c (red curve), underscoring its superior sensitivity to chiral interactions. As shown in Figure 6c (black curve), the optimized MDM-nanohole array structure exhibited a χ factor that shows similar behavior to the CD spectra, and which is approximately a factor of 10 larger than that of the single-layer gold nanohole array. Therefore, our simulations indicate that further enhancement can be possible, which renders this system as a highly promising candidate for ultrasensitive, enantioselective biosensing applications.

**Figure 6:**
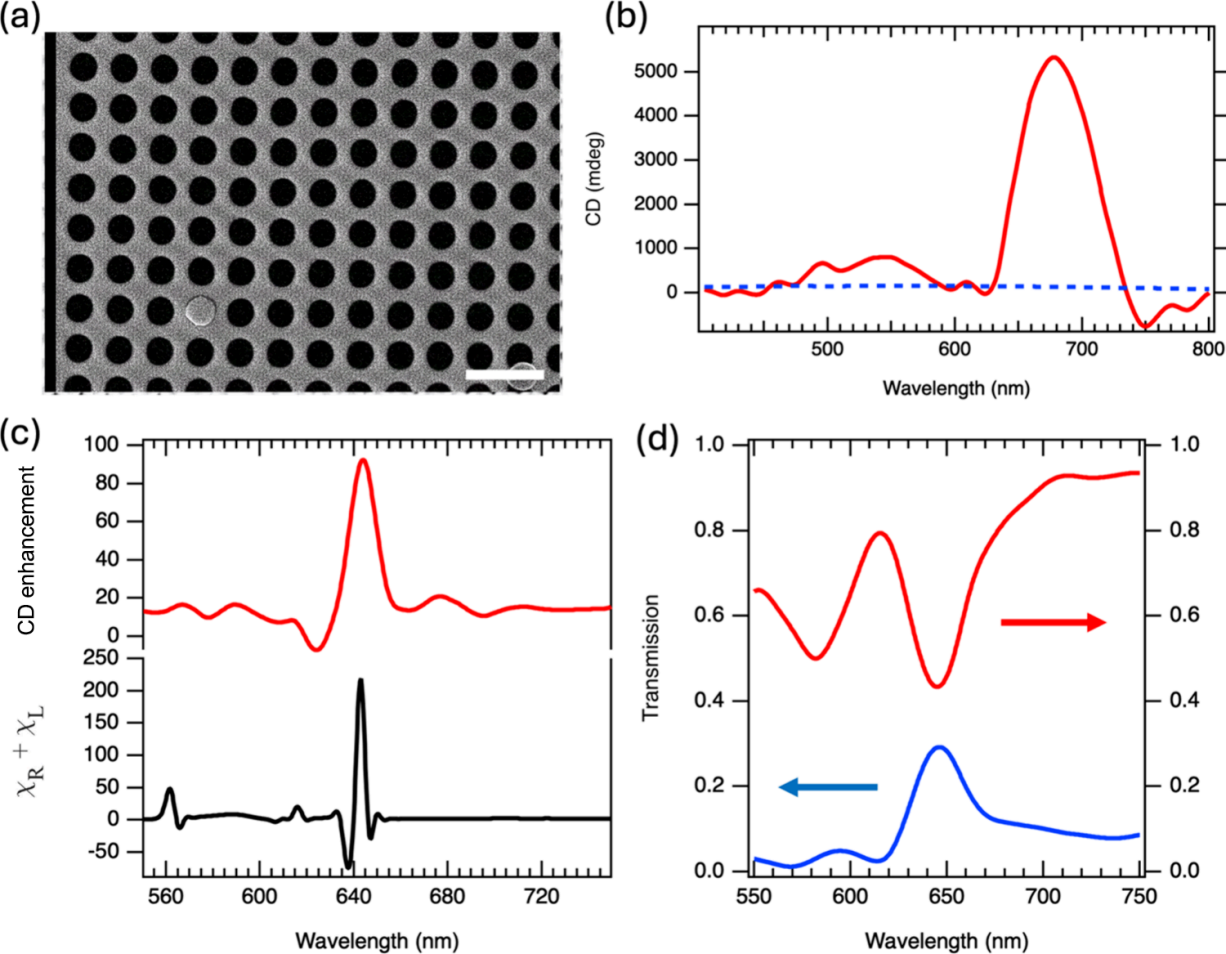
(a) SEM image of the MDM-nanohole array fabricated on a glass substrate, consisting of a 170 nm Al_2_O_3_ layer sandwiched between a 30 nm bottom Au layer and a 60 nm top Au layer with nanoholes. The nanoholes have a radius of 200 nm and are arranged in a lattice with a 530 nm constant. Scale bar is 1 μm. (b) Experimental CD spectra of l-phenylalanine deposited on the Au/Al_2_O_3_/Au nanohole array substrate (red curve) and on glass (blue-dashed line). A ∼50-fold enhancement in CD signal is observed for the Au/Al_2_O_3_/Au nanohole array configuration. (c) Simulated CD enhancement (red curve) for the Au/Al_2_O_3_/Au nanohole array structure, predicting up to an 80-fold amplification of the CD signal for l-phenylalanine. Averaged optical chirality enhancement factor χ (black curve) for the Au/Al_2_O_3_/Au nanohole array structure at a nanohole radius of 200 nm. (d) Simulated transmission and reflection spectra of the Au/Al_2_O_3_/Au nanohole array structure, showing a Fano resonance centered at 643 nm.

The transmission and reflection spectra of the MDM-nanohole array metasurface (Figure 6d) reveal a prominent resonance at λ = 643 nm. This feature aligns with peaks observed in both χ factor and CD response (Figure 6c). Based on its asymmetric profile, this resonance is attributed to a Fano resonance, which could arise from the interference between a broad surface lattice resonance (SLR) of the periodic gold nanohole array^37^ and a discrete Fabry-Pérot cavity mode within MDM nanohole architecture. This mutual coupling generates the characteristic Fano interference observed in the transmission spectra.^38,39^ The Fano resonance is well-known to generate enhanced near-field intensities and strong local electromagnetic confinement beyond what is achievable by isolated resonances, thereby amplifying optical effects at the nanoscale.^38,39^ The simultaneous spatial concentration and amplification of both electric and magnetic field components (evident in our finite element methods simulation, see Figure S4) markedly increase the local optical chirality density, which directly influences the differential transmission of right- and left-handed circularly polarized light through chiral molecules adsorbed on the surface.^20,40^ To quantify the resonance’s asymmetry and enhancement, we fitted the measured transmission spectra (Figure S5) with the standard Fano line shape function.^41^ and obtain an asymmetry factor *q* ≈ 3.3.^42^

We investigated the sensitivity of our MDM-nanohole array devices with CD measurements on L-PA films with different thicknesses, ranging from 20 to 140 nm (Figure S6a). To quantify the sensitivity we consider an operational spectral bandwidth of 680–700 nm, and normalize the integrated signal as CD_NORM_(*t*) = (CD_Film_(*t*) – CD_Substrate_)/CD_Substrate_ in this spectral range. The profile of CD_NORM_(*t*) resembles roughly a sigmoidal shape (Figure S6b), and we rationalize the saturation of the CD signal for large film thicknesses (exceeding 100 nm) with the increasing distance of the analyte molecules from the metasurface that reduces the efficiency of the signal enhancement. Based on this data set we can establish a detection limit of around 20 nm L-PA film thickness that corresponds to 10^8^ molecules in a spot size of 1 μm^2^.^44,45^ We further note that with control CD measurements using the achiral molecule rhodamine B we did not obtain any significant CD signal (Figure S7).

## Conclusions

We presented a comprehensive investigation on enhancing chiroptical responses using achiral nanostructures under oblique illumination. By introducing extrinsic chirality through tilted gold nanohole arrays, we achieved significant amplification of CD signals approximately by a factor of 20 in the presence of l-phenylalanine as a chiral biolayer compared to when it is placed on glass, which we obtained by both experimental measurements and numerical simulations.

We demonstrated that the χ factor is a key parameter in understanding and optimizing substrate-mediated chiroptical amplification. Building on these insights, we developed a multilayer Au/Al_2_O_3_/Au nanohole structure, which exhibited superior loop-formed current density distribution that boosts enhanced near-field interactions. The optimized MDM-nanohole array platform achieved a nearly 10-fold increase in the χ factor and a factor 2.5 increase in CD signal compared to single-layer gold nanohole arrays. This MDM-nanohole array substrate reached a 50-fold enhancement for l-phenylalanine, highlighting its potential for ultrasensitive, enantioselective biosensing applications.

Our findings establish extrinsic chirality engineering and χ factor optimization as powerful strategies for advancing plasmonic chiral sensors. This approach paves the way for the development of next-generation nanophotonic platforms with high sensitivity and selectivity for enantiomer detection.

## Methods

### Fabrication of Single-Layer Gold Nanohole Arrays Substrate

The nanohole arrays were fabricated on a clean fused silica substrate (12 × 12 mm^2^) using electron beam lithography (EBL). Initially, a positive electron-beam resist (PMMA, 950k A4) approximately 180 nm thick was spin-coated at 4000 rpm for 60 s and subsequently baked at 180 °C for 5 min. The EBL exposure was performed using an acceleration voltage of 20 kV, a beam current of 45 pA, and an exposure dose of 120 μC/cm^2^, with a writing speed of nearly 70 μm^2^/s, to pattern arrays covering a 200 × 200 μm^2^ area featuring nanohole diameters of 400 nm and a lattice constant of 530 nm. Following exposure, the resist was developed in a MIBK:IPA (1:3) solution for 60 s, rinsed with IPA, and dried under nitrogen. A conductive aluminum layer was locally deposited, and after aluminum removal, gold was deposited via electron beam evaporation at a rate of 0.6 Å/s onto the patterned substrate. Finally, the PMMA layer was removed using an ultrasonic acetone bath. The corresponding SEM image of the gold nanohole array substrate is shown in Figure 1a.

As a chiral biolayer, the l-phenylalanine biolayer was deposited using thermal evaporation of l-phenylalanine powder (density 1.34 g/cm^3^) at a controlled deposition rate of approximately 0.3 Å/s. The deposition was carried out at a substrate temperature of about 180 °C under a high vacuum environment maintained at approximately 5 × 10^–5^ mbar, conditions carefully maintained to promote the formation of a homogeneous layer. The film thickness was monitored using a quartz crystal microbalance based sensor (model SQC-330) in the thermal evaporator, which measures deposition rate and film thickness via quartz crystal microbalance. Atomic force microscopy (AFM) was employed to assess the uniformity of the approximately 60 nm thick film, confirming minimal thickness variation and consistent surface morphology across multiple locations on the substrate, as shown in the AFM image in Figure S3.

The fabrication protocol closely followed the method outlined in the referenced study,^10^ which utilized a similar thermal evaporation technique under comparable conditions. As shown in Figure S3d, the CD spectra of the deposited l-phenylalanine layer align well with literature values, indicating a reproducible and uniform molecular film. Additionally, optical transmission measurements of the bare l-phenylalanine film, displayed in Figure S3c, were recorded for s- and p-polarized light over the visible wavelength range, displaying a nearly flat profile without significant peaks or dips. Such a transmission signature indicates a uniform, continuous film with negligible scattering or inhomogeneities.

Thermal evaporation of l-phenylalanine was conducted at moderate temperatures (∼100 °C), well below its thermal decomposition onset (∼157–210 °C), preserving molecular integrity during deposition. Thermogravimetric and calorimetric studies in the literature confirm l-phenylalanine’s thermal stability below these temperatures. During CD measurements, illumination intensities and substrate temperatures were carefully controlled to avoid thermal or photochemical degradation, ensuring the photostability of the solid film.

### Fabrication of Au/Al**_2_** O**_3_**/Au Structure

A 12 × 12 mm^2^ fused silica substrate was first coated with a 5 nm titanium adhesion layer, followed by deposition of a 30 nm gold layer and a 170 nm Al_2_O_3_ dielectric spacer via electron beam evaporation with a rate of 0.5 and 1 Å/s for the gold layer and dielectric spacer, respectively. A PMMA layer was then spin-coated on top of the Al_2_O_3_ surface, and nanohole arrays were patterned using EBL under the same conditions described above. After the development step, a 60 nm gold top layer was deposited, and lift-off was performed in an acetone bath. The resulting structure consisted of nanohole arrays embedded in the top gold layer, forming the final Au/Al_2_O_3_/Au trilayer configuration, as shown in Figure 5a. An SEM image of this configuration is displayed in Figure 6a. The l-phenylalanine biolayer (60 nm thick) was deposited onto the Au/Al_2_O_3_/Au structure via thermal evaporation, following the same procedure as for the single-layer sample.

### Finite Element Method Simulations

The model consisted of a nanohole array on a gold-silica substrate, with a lattice constant of 530 nm, a gold layer thickness of 80 nm, and a nanohole diameter of 400 nm. The unit cell was modeled using periodic boundary conditions. For the mode analysis study, an effective refractive index model was employed. Excitation was applied using ports placed on the top and bottom surfaces of the model, with electric field amplitudes defined for right- and left-handed circular polarization based on the Jones matrix. The refractive index used for the materials involved in the simulations are shown in Figure S8a (Al_2_O_3_) and Figure S8b (Au) and were experimentally retrieved by spectroscopic ellipsometry. The desired angle of incidence, defined by θ and φ, was specified in the port settings under the Elevation and Azimuth angle of incidence input fields. The simulation was solved over a frequency range within the visible spectrum using a Parametric Sweep to explore the resonance range of the gold nanohole array. Mesh refinement was applied around the nanoholes to ensure accuracy in field distribution calculations. The results focused on transmission spectra and field distributions, highlighting the effect of circular polarization on the system’s optical response. The simulation results were compared with experimentally recorded data, validating the accuracy and reliability of the model.

## Supplementary Material



## Notes

Optical setup for circular dichroism; characterization of l-phenylalanine thin films; transmission of planar Au/Al_2_O_3_/Au multilayers; transmission spectrum and Fano resonance; circular dichroism experiments of L-PA films with different thickness, and with achiral rhodamine B molecules; and refractive index and extinction coefficient spectra of Au and Al_2_O_3_ films measured by spectroscopic ellipsometry (PDF)
