# Supplementary material for: Ultrasensitive and Tunable Achiral Metamaterial Substrates as Nanobiosensors for Enantiomer Detection
Source: ACS Appl Mater Interfaces. 2025 Nov 13;17(47):65270–80. doi: 10.1021/acsami.5c14316 (PMC12673517; doi:10.1021/acsami.5c14316)
Supplement: Supplementary file 1 [file am5c14316_si_001.pdf]

Supporting Information

Ultra-Sensitive and Tunable Achiral Metamaterial Substrates as Nanobiosensors for Enantiomer Detection

Maryam Mirahmadi,*,†,‡ Ali Douaki,†,¥ Vincenzo Caligiuri,¶,§ Denis Garoli,†,¥ and Roman Krahne*,†

†Optoelectronics, Istituto Italiano di Tecnologia (IIT), via Morego 30, 16163 Genova, Italy.

¥Dipartimento di Science e Metodi per l'Ingegneria, Università degli Studi di Modena e Reggio Emilia, Viale Amendola 2, 14122, Reggio Emilia (Italy)

‡Department of Chemistry and Industrial Chemistry, University of Genoa, via Dodecaneso 31, 16146 Genova, Italy.

¶Dipartimento di Fisica, Universit`a della Calabria, via P. Bucci 33b, 87036 Rende (CS), Italy.

§Consiglio Nazionale delle Ricerche – Istituto di Nanotecnologia (CNR-Nanotec), via P. Bucci 33c, 87036 Rende, Italy.

E-mail: maryam.mirahmadi@iit.it; roman.krahne@iit.it

1. Optical Setup for Circular Dichroism

The optical setup for CD measurements, as shown in Figure S1, includes a xenon lamp as a broadband light source. The emission is first collimated and then passes through a quarter-wave plate to generate circularly polarized light. The beam is focused onto the sample using an objective lens, with the sample mounted on a rotational stage to allow control of incidence angles. Transmitted light is collected by a second objective and directed to a spectrometer for spectral analysis (400–800 nm range). A CMOS camera is included in the setup to facilitate precise alignment of the patterned nanohole area with the incident light during measurements.

The numerical aperture of the microscope objective used is 0.4, which limits the maximum permissible tilt angle for full light collection to approximately $\theta_{\max} = \arcsin\left(\frac{\mathrm{NA}}{n}\right) \approx 23.6°$. However, practical considerations in our optical setup restricted the experimental tilt angles to around 10° to ensure accurate and efficient light collection within this angular range.

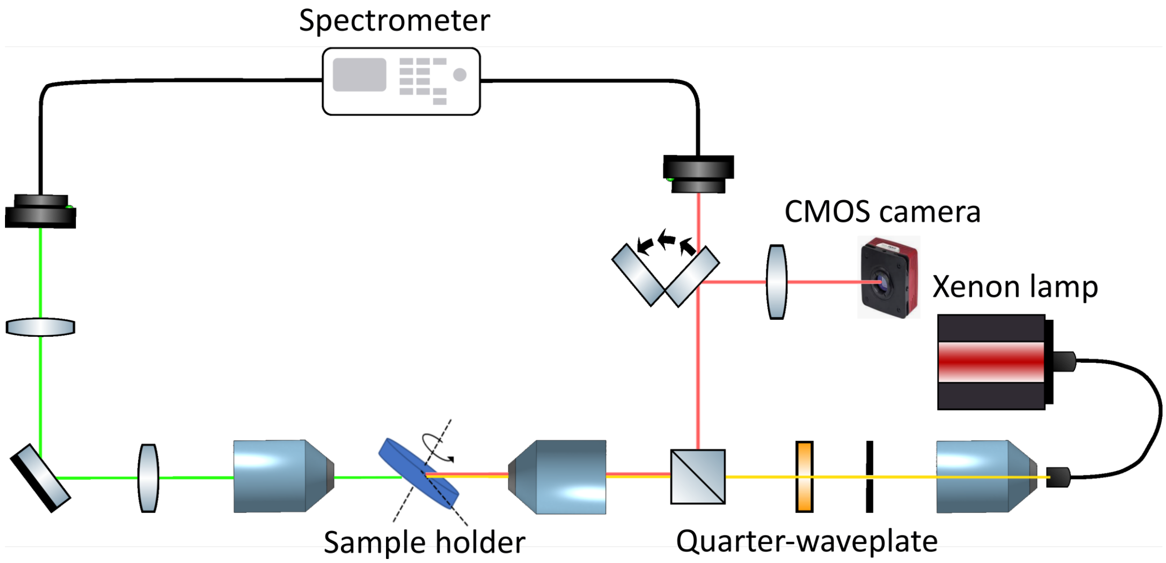

Figure S1. Schematic of the optical setup used for CD measurements.

2. Characterization of L-Phenylalanine Thin Films

The morphology and thickness of the thermally evaporated L-phenylalanine (LPA) thin film on glass were characterized using AFM. Figure S2a presents a representative AFM topographic image, revealing the overall surface characteristics. A line profile, shown in Figure S2b, was extracted across a defined step on the film, confirming an average thickness of approximately 60 nm. The AFM analysis substantiates the formation of a continuous L-phenylalanine layer, suitable for its intended function as a chiral biolayer. The optical and chiroptical properties of the bare L-phenylalanine film were further evaluated. Figure S2c shows the high transmission (>96%) of the LPA film on glass under s- and p-polarized light across the visible spectrum. Figure S2d displays the corresponding circular dichroism spectrum, revealing the intrinsic chiral response of the evaporated LPA layer on glass.

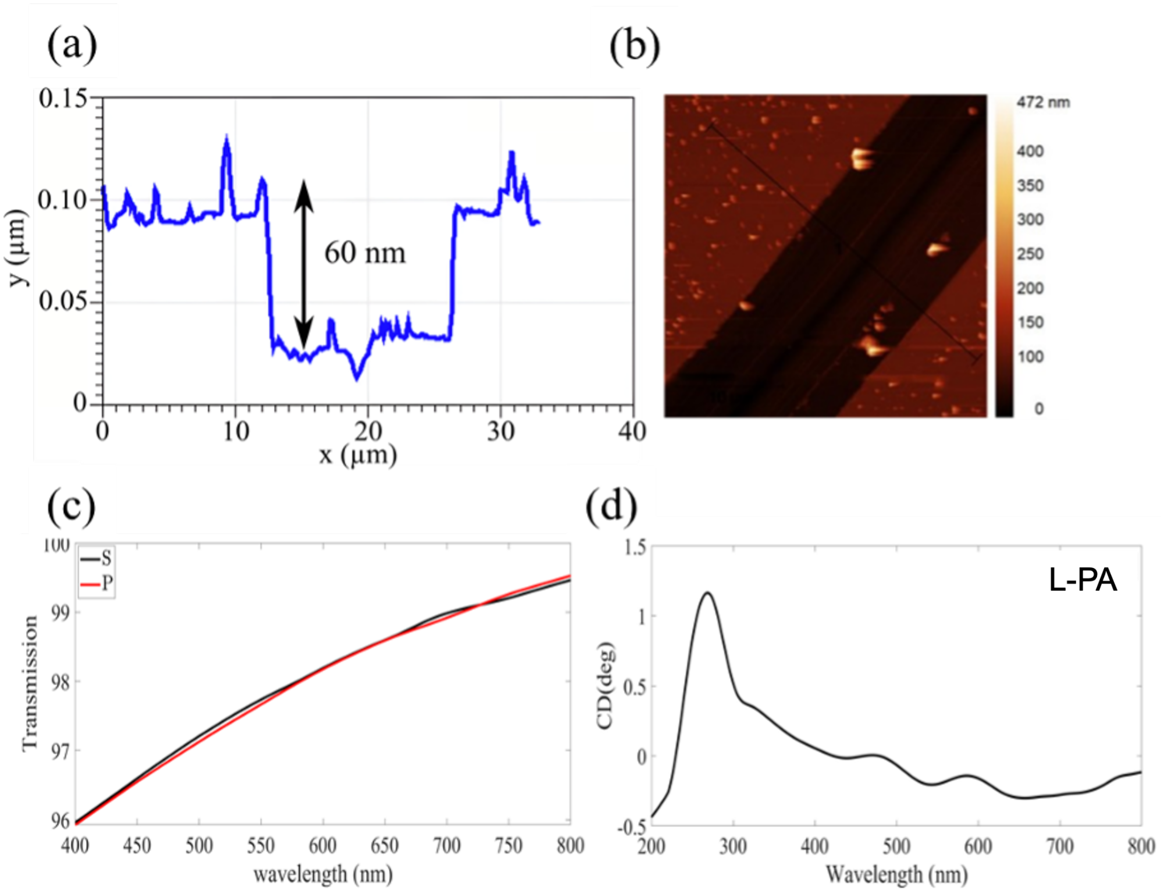

Figure S2. Atomic Force Microscopy (AFM) image (a) and corresponding height profile (b) of the thermally evaporated L-phenylalanine thin film on a glass substrate. (c) Optical transmission spectra for bare L-phenylalanine (L-PA) film deposited on glass, measured under s- and p-polarized illumination, showing high and transparency across the 400–800 nm range exhibit regular features. (d) Corresponding CD spectrum of glass–LPA films, illustrating intrinsic chiroptical response of L-phenylalanine in the absence of plasmonic structures.

3. Transmission of Planar Au/Al_2O_3/Au Multilayers

To isolate the contribution of the nanohole array to the optical response, we compared the transmission spectra of the Au/Al_2O_3/Au MDM structure with nanoholes (Figure 1) to a planar reference without nanoholes (Figure S2). The introduction of nanoholes induces a notable spectral shift of the maximum transmission resonances**,** particularly evident as a blue shift around 170 nm thickness (e.g., from ~700 nm in the planar structure to 643 nm with nanoholes). Furthermore, the nanohole array generates additional spectral features that are not present in the planar MDM structure. These new resonances arise from the coupling between localized surface plasmon modes supported by individual nanoholes and the guided modes of the MDM waveguide structure [1]. The enhanced transmission intensity observed in the nanohole array, visible in the color bar legends, corresponds to the phenomenon of extraordinary optical transmission (EOT) [2], where the periodic nanohole pattern enables efficient light coupling through subwavelength apertures

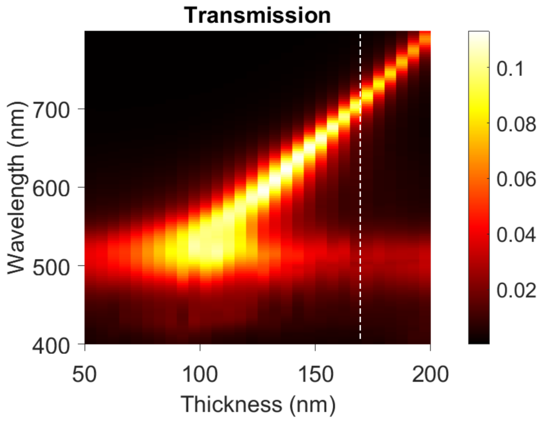

Figure S3. Transmission spectrum of the planar Au/Al_2O_3/Au multilayer thin film as a function of wavelength and Al_2O_3 thickness.

4. Transmission Spectrum and Fano Resonance

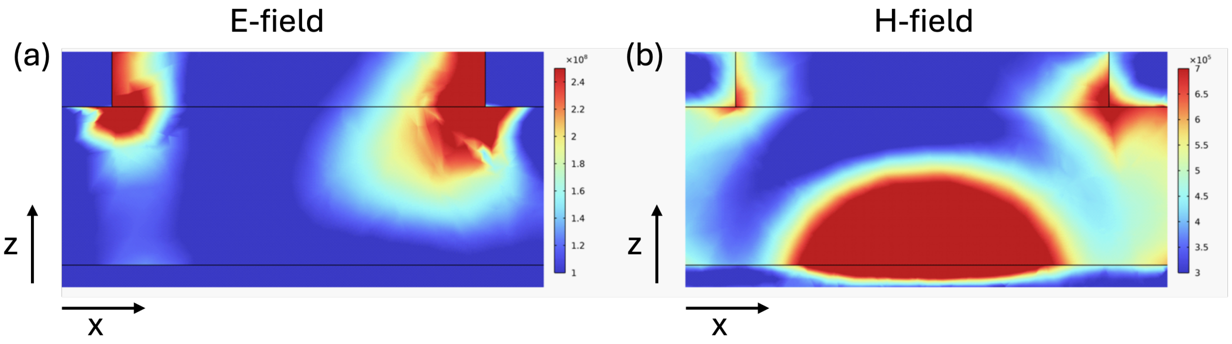

Figure S4. Electric (a) and magnetic (b) field hot spots in the nanohole array – MDM structure calculated by finite element methods simulations for λ = 643 nm. The tilting angle is 10°.

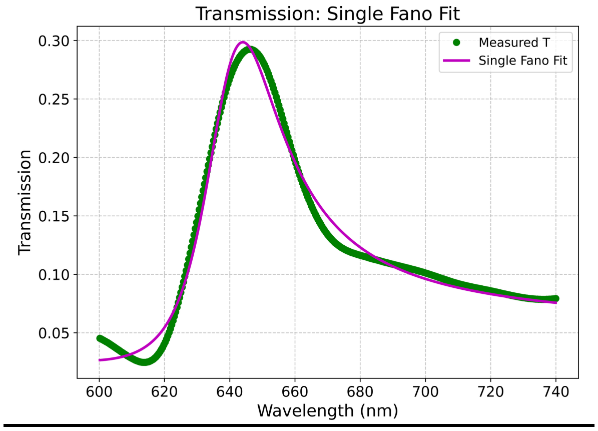

Figure S5. Measured transmission spectrum of the nanostructure with corresponding fit using the standard Fano line shape function [3].

5. **<u>Circular dichroism experiments with different film thicknesses of L-PA and achiral molecule rhodamine B.</u>**

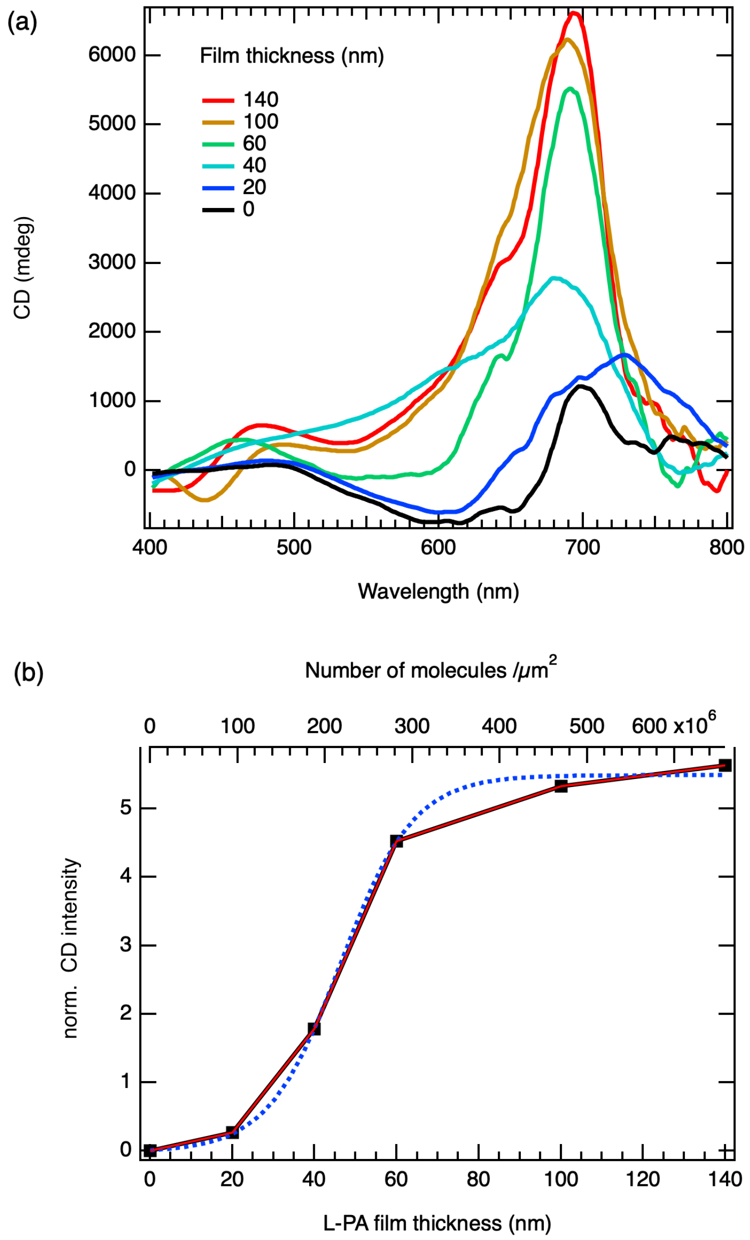

Figure S6. (a) CD spectra for L-phenylalanine (L-PA) films with different thickness in the range from 20-140 nm. (b) CD enhancement versus film thickness, and number of molecules in a 1μm spot region. The dashed blue line shows a sigmoidal fit to the data.

The normalized CD intensity is calculated as $CD_{NORM}(t) = (CD_{Film}(t) - CD_{Substrate})/CD_{Substrate}$ in the spectral range of 680-700 nm.

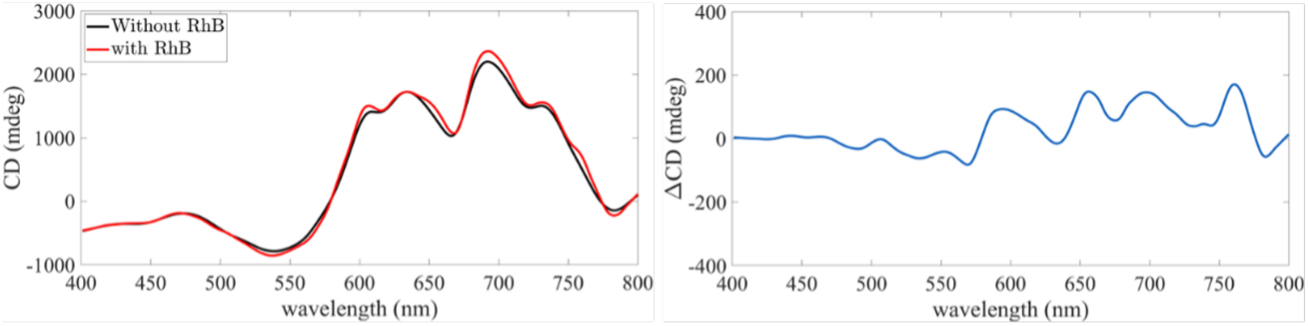

Figure S7. CD measurements of rhodamine B as non-chiral molecule. Left: Comparative CD spectra for MDM-nanohole array structure and achiral rhodamine B coatings. Right: Differential CD response (ΔCD) between structure with and without rhodamine B and sensor, indicating negligible enhancement for non-chiral species and validating the specificity of the platform.

6. Refractive Index and Extinction Coefficient Spectra of Au and Al_2O_3 Films Measured by Spectroscopic Ellipsometry

The refractive index used for the materials involved in the simulations are shown in Figure S8a (Al_2O_3) and Figure S8b (Au) and were experimentally retrieved by spectroscopic ellipsometry.

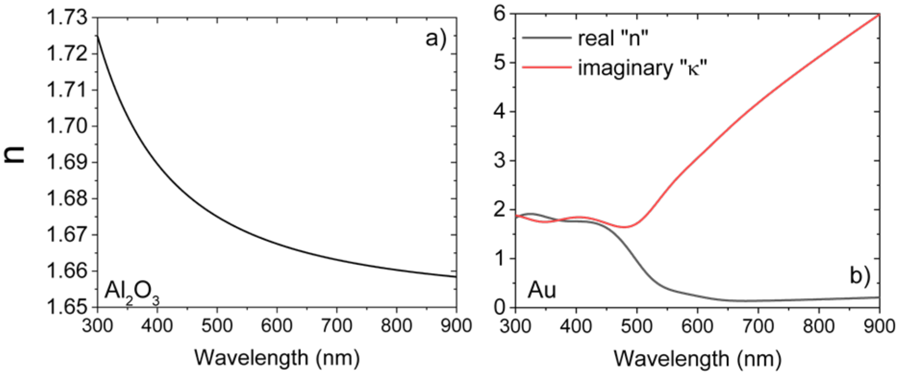

Figure S8. Refractive index of the Al_2O_3 and Au films used in the simulations.

References:

[1]: Wong, Y. L., Jia, H., Jian, A., Lei, D., El Abed, A. I., & Zhang, X. (2021). Enhancing plasmonic hot-carrier generation by strong coupling of multiple resonant modes. *Nanoscale*, *13*(5), 2792-2800.

[2]: Ebbesen, T. W., Lezec, H. J., Ghaemi, H. F., Thio, T., & Wolff, P. A. (1998). Extraordinary optical transmission through sub-wavelength hole arrays. *nature*, *391*(6668), 667-669.

[3]: Limonov, M. F., Rybin, M. V., Poddubny, A. N., & Kivshar, Y. S. (2017). Fano resonances in photonics. Nature photonics, 11(9), 543-554.
